# Cell Surface GRP78 as a Death Receptor and an Anticancer Drug Target

**DOI:** 10.3390/cancers11111787

**Published:** 2019-11-13

**Authors:** Ruowen Ge, Chieh Kao

**Affiliations:** Department of Biological Sciences, National University of Singapore, Singapore 117558, Singapore; ekingo@gmail.com

**Keywords:** cell surface GRP78 (csGRP78), death receptor, apoptosis, anticancer drug

## Abstract

Cell surface GRP78 (csGRP78, glucose-regulated protein 78 kDa) is preferentially overexpressed in aggressive, metastatic, and chemo-resistant cancers. GRP78 is best studied as a chaperone protein in the lumen of endoplasmic reticulum (ER), facilitating folding and secretion of the newly synthesized proteins and regulating protein degradation as an ER stress sensor in the unfolded protein pathway. As a cell surface signal receptor, multiple csGRP78 ligands have been discovered to date, and they trigger various downstream cell signaling pathways including pro-proliferative, pro-survival, and pro-apoptotic pathways. In this perspective, we evaluate csGRP78 as a cell surface death receptor and its prospect as an anticancer drug target. The pro-apoptotic ligands of csGRP78 discovered so far include natural proteins, monoclonal antibodies, and synthetic peptides. Even the secreted GRP78 itself was recently found to function as a pro-apoptotic ligand for csGRP78, mediating pancreatic β-cell death. As csGRP78 is found to mainly configur as an external peripheral protein on cancer cell surface, how it can transmit death signals to the cytoplasmic environment remains enigmatic. With the recent encouraging results from the natural csGRP78 targeting pro-apoptotic monoclonal antibody PAT-SM6 in early-stage cancer clinical trials, the potential to develop a novel class of anticancer therapeutics targeting csGRP78 is becoming more compelling.

## 1. Introduction

Glucose-regulated protein 78 kDa (GRP78), also referred to as HSPA5 (heat shock 70 kDa protein 5) and BiP (immunoglobulin heavy-chain binding protein), was first discovered and characterized as an endoplasmic reticulum (ER) resident protein [1,2]. The traditional function of GRP78 is a molecular chaperone in the ER lumen, helping to regulate protein quality control, facilitating protein folding, assembly, and misfolded protein degradation in the unfolded protein response (UPR) pathway [3]. GRP78 serves as a major ER stress sensor and is upregulated under ER stress, helping to maintain ER homeostasis and cell survival. In cancer, GRP78 is significantly upregulated due to the highly stressful microenvironment of cancer, serving as a pro-survival and anti-apoptotic protein for cancer cells [4].

In addition to function as an ER chaperon and stress sensor, GRP78 is also found in other sub-cellular locations such as on the cell surface or secreted into the extracellular environment. Cell surface GRP78 (csGRP78) functions as an important signal receptor, transmitting signals from the extracellular environment into cells [5]. To date, several ligands have been discovered to interact with csGRP78, including secreted proteins and plasma membrane-anchored proteins. Through interactions with these ligands, csGRP78 activates multiple intracellular cell signaling pathways, impacting cell proliferation, survival, migration, or apoptosis. Various pro-proliferative, pro-survival ligands, and pro-apoptotic ligands have been discovered, including natural proteins, monoclonal antibodies (Mabs), and synthetic peptides, even the secreted extracellular GRP78 itself [6]. In addition to extracellular ligands, several plasma membrane-bound proteins have also been demonstrated to interact with csGRP78, such as the glycosylphosphatidylinositol-anchored (GPI-anchored) proteins Cripto, T-cadherin, and CD109 [7,8,9].

Due to its preferential presence on the cell surface of cancer cells, csGRP78 has emerged as an attractive target for anticancer drugs [4]. Many excellent previous reviews have presented the diverse roles of GRP78 in multiple subcellular locations, and the different functions that GRP78 plays in cancer as well as other diseases [5,10,11,12,13,14,15,16,17,18]. However, the role of csGRP78 as a cell surface death receptor has not been comprehensively evaluated. In this perspective, we focus on csGRP78 as a death receptor and discuss its significance as a target for proapoptotic ligand-mediated anticancer drug development.

## 2. csGRP78 as a Death Receptor

**Figure 1:**
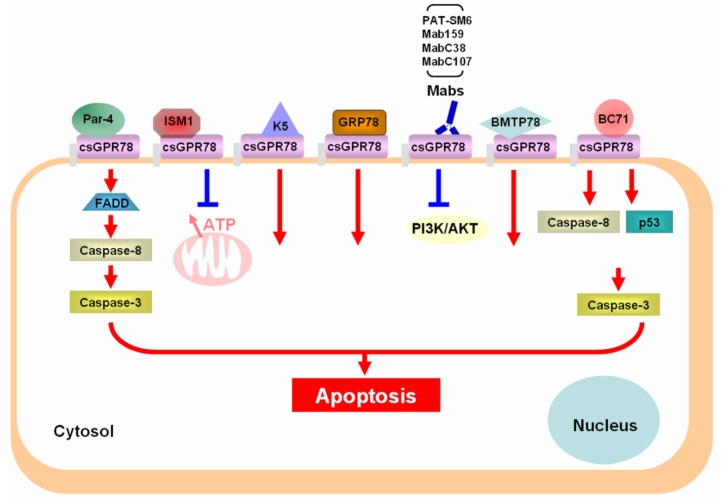
Summary of the pro-apoptotic ligands of csGRP78 and their mechanism of action. Par-4 (Prostate Apoptosis Response-4, ISM1 (Isthmin 1), K5 (plasminogen Kringle 5), Mabs (monoclonal antibodies), FADD (Fas associated protein with death domain), PI3K (PI3 kinase).

The classical death receptors are members of the tumor necrosis receptor superfamily characterized by the presence of a cytoplasmic death domain, which is critical for the death receptor to initiate downstream cytotoxic signaling pathways involving caspases [19]. However, csGRP78 has been shown to be a predominantly external peripheral protein on the plasma membrane in several cultured cancer cell lines, with no transmembrane and cytosolic domain present [20]. A substantial level of csGRP78 achieved plasma membrane localization by interacting with GPI-anchored proteins. A membrane embedded form of csGRP78 was shown to be present only under ER stress conditions in these cancer cells, and at a very low level. Hence, how csGRP78 functions as a death receptor to transmit extracellular death signals to intracellular cytotoxic signaling pathways is intriguing and remains largely unknown. The known pro-apoptotic ligands of csGRP78, including natural proteins, monoclonal antibodies, and synthetic peptides, are summarized in Figure 1.

## 3. Natural Proapoptotic Protein Ligands of csGRP78

To date, at least four naturally secreted proteins have been shown to function as proapoptotic ligands of csGRP78, triggering cell death signaling (Figure 1). 

### 3.1. Prostate Apoptosis Response-4 (Par-4)

**Figure 2:**
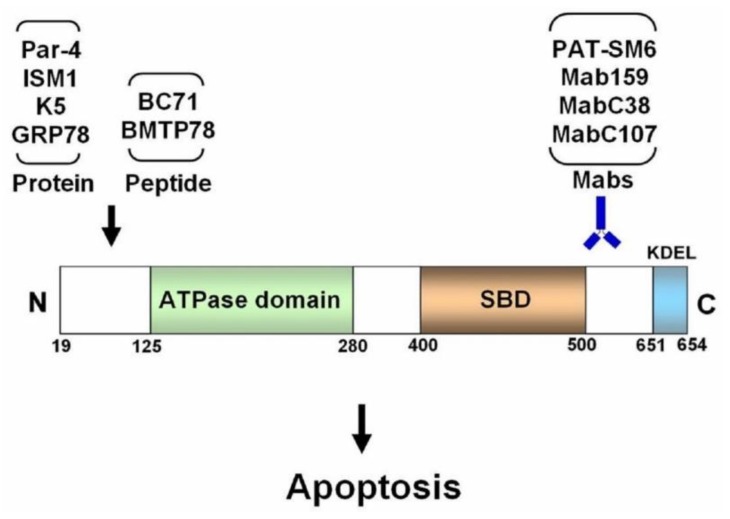
Schematic illustration of the region of human GRP78 that the various proapoptotic ligands interact with. SBD: substrate binding domain, KDEL: the 4 residue ER retention signal at the C-terminus of GRP78. The amino acid boundary of the domains are labelled at the bottom of the GRP78 protein.

A well-studied proapoptotic ligand of csGRP78 is the secreted prostate apoptosis response-4 (Par-4) protein [21]. Par-4 is expressed in various tissues and was first identified as a tumor suppressor localized in the cytosol and nucleus. It promotes apoptosis through the mitochondrial mediated intrinsic apoptotic pathway [22]. Subsequently, Par-4 is found to be secreted into the extracellular environment by both cancer cells and normal cells. Extracellular Par-4 functions as a proapoptotic protein, selectively targeting csGRP78 on cancer cells to trigger cancer-specific apoptosis via its SAC (selective for apoptosis induction in cancer cells) domain. Par-4 induces apoptosis by recruiting and activating the adaptor protein, Fas-associated protein with death domain (FADD), leading to downstream caspase-8 activation [21,23]. Moreover, apoptosis induced by the death ligand TRAIL (TNF-related apoptosis-inducing ligand) is dependent on extracellular Par-4 signaling via csGRP78. Notably, Par-4 interacts with the N-terminal region of csGRP78 (Figure 2). Systemic application of recombinant Par-4 or its proapoptotic domain SAC potently inhibited tumor growth in mice [21,24]. 

### 3.2. Isthmin 1 (ISM1)

Isthmin 1 (ISM1) is a secreted 70 kDa protein (theoretical molecular weight 50 kDa) in vertebrates. It was first identified by our lab as an angiogenesis inhibitor, inducing apoptosis in endothelial cells via αvβ5 integrin as a soluble protein [25]. However, in a surface-anchored form, ISM1 support endothelial cell adhesion and survival instead [26]. Subsequently, csGRP78 was identified as a high-affinity receptor for ISM1, and ISM1/csGRP78 interaction triggers apoptosis in both activated endothelial cells and cancer cells that harbor high levels of csGRP78 [27]. Interestingly, ISM1 also interacts with the N-terminal region of csGRP78, similar to Par-4 (our unpublished result, Figure 2). ISM1/csGRP78 interaction lead to the internalization of ISM1 via clathrin-mediated endocytosis and the trafficking of ISM1 to mitochondria, resulting in mitochondria dysfunction by blocking ATP/ADP exchange on the mitochondrial membrane [28]. The decline of cytosolic ATP concentration eventually caused apoptosis. Systemic infusion of recombinant ISM1 via intravenous route potently suppressed xenograft tumor growth in mice [27].

### 3.3. Plasminogen Kringle 5 (K5)

Plasminogen kringle 5 (K5) is a natural proteolytic fragment of the blood protein plasminogen, containing its fifth kringle domain. It functions as an angiogenesis inhibitor, inducing apoptosis of endothelial cells [29]. K5 was identified as a ligand for csGRP78, binding to the N-terminal domain of GRP78 [30,31] (Figure 2). It abrogates cell migration and trigger apoptosis via csGRP78 on endothelial cells and cancer cells. Anti-GRP78 antibody targeting the N-terminal region of GRP78 attenuated K5-induced inhibition of endothelial cell migration. Vaspin (visceral adipose tissue-derived serine proteinase inhibitor), an adipokine, was identified as a novel high-affinity ligand of csGRP78 that competes with K5 for csGRP78 binding and antagonize K5 function. Vaspin dose-dependently suppressed K5-induced intracellular Ca^2+^ influx and subsequent apoptosis in endothelial cells [32]. Recently, K5 was shown to dose-dependently downregulate GRP78 expression in gastric cancer cells. Downregulation of GRP78 contributes to K5-induced apoptosis in gastric cancer cells [33].

### 3.4. Secreted GRP78

GRP78 is known to also exist as a secreted soluble protein in the extracellular environment and in the serum [5]. Recently, extracellular GRP78 itself was identified as a proapoptotic ligand of csGRP78, triggering caspase-mediated apoptosis in stressed pancreatic beta cells [6]. Pro-inflammatory cytokines induce ER stress in beta cells, leading to the secretion and plasma membrane translocation of GRP78 [34]. csGRP78 was shown to serve as a death receptor for the secreted extracellular GRP78 which serves as a self-ligand to activate a proapoptotic pathway in these cells, leading to cell death. In addition, recombinant GRP78 induced apoptosis in cytokine-treated beta cells, but not in untreated control cells. Anti-GRP78 antibody targeting both N- and C-terminal regions of GRP78 blocked extracellular GRP78-induced apoptosis. These results suggest a possible pathway of active self-destruction in cytokine-exposed pancreatic beta cells mediated through GRP78, with soluble extracellular GRP78 activating csGRP78 for this self-destruction. csGRP78 may be an important modulator of beta cell death upon inflammatory stress responses and a therapeutic target for type I diabetes.

## 4. Monoclonal Antibodies as Proapoptotic Ligands of csGRP78

Different anti-GRP78 Mabs generate different biological consequences in the target cells depending on the Mab and the particular region of GRP78 it targets. The consequences of a Mab binding to csGRP78 include stimulation of cell proliferation, suppression of cell proliferation, triggering apoptosis, or no effect at all. It remains unclear why different anti-GRP78 Mabs generate different biological effects in cells. Previously, it has been postulated that an antibody targeting the C-terminal region of GRP78 may be a pan suppressor of proliferative/survival signaling of csGRP78 in cancer cells. However, not all antibodies against the C-terminal region of GRP78 present suppressive activity in cancer [35,36]. The GRP78 C-terminal targeting Mabs C38 and C107 both significantly suppressed tumor growth in prostate and melanoma models by activating caspase-mediated apoptosis [37,38]. A mouse Mab targeting the KDEL ER retention sequence at the C-terminus of GRP78 also inhibited cell proliferation and induced apoptosis [39]. However, a human Mab targeting the C-terminal 20 residues affected neither cell proliferation nor apoptosis [40]. Mab159, a GRP78-specific mouse monoclonal IgG, suppressed multiple types of cancer growth and metastasis in mice by inducing cancer cell apoptosis and suppressing PI3K/AKT signaling [41]. The GRP78 region targeted by Mab159 is also towards the C-terminal region [42] (Figure 2). PAT-SM6, a human monoclonal IgM isolated from a gastric cancer patient, induced apoptosis of multiple myeloma and melanoma cells in vitro and suppressed cancer growth in xenograft models [43,44]. PAT-SM6 recognized an O-linked carbohydrate moiety of csGRP78 with a molecular weight of 82 kDa, specifically present in cancer cells [45]. Nevertheless, to date, no report has demonstrated that csGRP78 is of a different protein composition, comparing to the ER lumen GRP78. csGRP78 expression is known to increase with the progression of multiple myeloma and is highly elevated in multiple myeloma patients presenting drug-resistant and extramedullary disease phenotypes. About one-third of multiple myeloma patients with relapsed or refractory disease showed stability after two weeks of PAT-SM6 treatment in a phase I clinical trial [46]. PAT-SM6 in combination with bortezomib and lenalidomide leads to partial remission of both intra- and extramedullary lesions in a patient with drug-resistant multiple myeloma [47].

## 5. Synthetic Peptides as Proapoptotic Ligands of csGRP78

Several synthetic peptides able to induce apoptosis by targeting the N-terminal region of GRP78 have been developed (Figure 2). Peptides WIFPWIQL and WDLAWMFRLPVG were selected by phage-binding assays for their ability to bind GRP78 specifically [48]. When these two GRP78-binding peptides were fused with a proapoptotic peptidomimetic, the two resulting peptides, WIFPWIQL-GG-_D_(KLAKLAK)_2_ (later called BMTP78) and WDLAWMFRLPVG-GG-_D_(KLAKLAK)_2_, both selectively targeted tumors and suppressed tumor growth in vivo by inducing cancer cell apoptosis. BMTP78 selectively killed breast cancer cells that expressed csGRP78 and suppressed both primary tumor growth as well as lung and bone micrometastases, leading to prolonged disease-free survival [49]. Despite the promising antitumor effect of BMTP-78 in vitro and in preclinical cancer models, subsequent toxicology studies in nonhuman primates presented unexpected cardiac toxicity, leading to morbidity and mortality [50]. This cardiac toxicity reduced the optimism for BMTP78 to become an anticancer drug. Recently, BC71, a cyclic pentapeptide derivative of ISM1, was shown to bind specifically to GRP78 and trigger apoptosis in cultured endothelial cells [51]. BC71 has the unique ability to both bind to csGRP78 and trigger apoptosis by itself. In vivo, BC71 preferentially accumulated in the tumor and suppressed xenograft tumor growth in mice. Hence, BC71 can be useful as a prototype peptide to develop further modified peptides with higher GRP78-binding affinity and more potent proapoptotic activity for anticancer drug development. 

## 6. Conclusions and Future Perspectives

Due to the preferential presence of csGRP78 on cancer cells, csGRP78 has emerged as an attractive target for anticancer drug development. Proapoptotic ligands of csGRP78, including natural proteins, Mabs, and synthetic peptides, have the potential to become effective anticancer drugs. Although no anticancer drugs targeting csGRP78 have reached the clinic as of now, the Mab PAT-SM6 has shown promising results in early-stage clinical trials [46,47]. We envisage that a csGRP78-targeted proapoptotic anticancer drug is likely to emerge in the coming years. Nevertheless, how csGRP78 initiates the intracellular death signaling pathway remains poorly understood, especially because it sits on cancer cells preferentially as an external peripheral protein [20]. One hypothesis is that csGRP78 forms complexes with other cell surface transmembrane proteins to transmit the death signal to the intracellular environment. Future research in this interesting area is highly warranted. A more in-depth understanding of how csGRP78 functions as a cell surface signal receptor will greatly facilitate targeted cancer drug development.

## References

[1] Shiu R.P., Pouyssegur J., Pastan I. (1977). Glucose depletion accounts for the induction of two transformation-sensitive membrane proteinsin rous sarcoma virus-transformed chick embryo fibroblasts. Proc. Natl. Acad. Sci. USA.

[2] Zala C.A., Salas-Prato M., Yan W.T., Banjo B., Perdue J.F. (1980). In cultured chick embryo fibroblasts the hexose transport components are not the 75 000 and 95 000 dalton polypeptides synthesized following glucose deprivation. Can. J. Biochem..

[3] Lee A.S. (2001). The glucose-regulated proteins: Stress induction and clinical applications. Trends Biochem. Sci..

[4] Lee A.S. (2007). Grp78 induction in cancer: Therapeutic and prognostic implications. Cancer Res..

[5] Ni M., Zhang Y., Lee A.S. (2011). Beyond the endoplasmic reticulum: Atypical grp78 in cell viability, signalling and therapeutic targeting. Biochem. J..

[6] Vig S., Buitinga M., Rondas D., Crevecoeur I., van Zandvoort M., Waelkens E., Eizirik D.L., Gysemans C., Baatsen P., Mathieu C. (2019). Cytokine-induced translocation of grp78 to the plasma membrane triggers a pro-apoptotic feedback loop in pancreatic beta cells. Cell Death Dis..

[7] Shani G., Fischer W.H., Justice N.J., Kelber J.A., Vale W., Gray P.C. (2008). Grp78 and cripto form a complex at the cell surface and collaborate to inhibit transforming growth factor beta signaling and enhance cell growth. Mol. Cell Biol..

[8] Tsai Y.L., Ha D.P., Zhao H., Carlos A.J., Wei S., Pun T.K., Wu K., Zandi E., Kelly K., Lee A.S. (2018). Endoplasmic reticulum stress activates src, relocating chaperones to the cell surface where grp78/cd109 blocks tgf-beta signaling. Proc. Natl. Acad. Sci. USA.

[9] Philippova M., Ivanov D., Joshi M.B., Kyriakakis E., Rupp K., Afonyushkin T., Bochkov V., Erne P., Resink T.J. (2008). Identification of proteins associating with glycosylphosphatidylinositol- anchored t-cadherin on the surface of vascular endothelial cells: Role for grp78/bip in t-cadherin-dependent cell survival. Mol. Cell Biol..

[10] Quinones Q.J., de Ridder G.G., Pizzo S.V. (2008). Grp78: A chaperone with diverse roles beyond the endoplasmic reticulum. Histol. Histopathol..

[11] Gonzalez-Gronow M., Selim M.A., Papalas J., Pizzo S.V. (2009). Grp78: A multifunctional receptor on the cell surface. Antioxid. Redox Signal..

[12] Sato M., Yao V.J., Arap W., Pasqualini R. (2010). Grp78 signaling hub a receptor for targeted tumor therapy. Adv. Genet..

[13] Pizzo S.V., Pizzo S.V. (2018). Cell Surface Grp78, a New Paradigm in Signal Transduction Biology.

[14] Ibrahim I.M., Abdelmalek D.H., Elfiky A.A. (2019). Grp78: A cell’s response to stress. Life Sci..

[15] Gifford J.B., Hill R. (2018). Grp78 influences chemoresistance and prognosis in cancer. Curr. Drug Targets.

[16] Casas C. (2017). Grp78 at the centre of the stage in cancer and neuroprotection. Front. Neurosci..

[17] Cook K.L., Clarke R. (2015). Role of grp78 in promoting therapeutic-resistant breast cancer. Future Med. Chem..

[18] Bailly C., Waring M.J. (2019). Pharmacological effectors of grp78 chaperone in cancers. Biochem. Pharmacol..

[19] Guicciardi M.E., Gores G.J. (2009). Life and death by death receptors. FASEB J..

[20] Tsai Y.L., Zhang Y., Tseng C.C., Stanciauskas R., Pinaud F., Lee A.S. (2015). Characterization and mechanism of stress-induced translocation of 78-kilodalton glucose-regulated protein (grp78) to the cell surface. J. Biol. Chem..

[21] Burikhanov R., Zhao Y., Goswami A., Qiu S., Schwarze S.R., Rangnekar V.M. (2009). The tumor suppressor par-4 activates an extrinsic pathway for apoptosis. Cell.

[22] Irby R.B., Kline C.L. (2013). Par-4 as a potential target for cancer therapy. Expert Opin. Ther. Targets.

[23] Lee A.S. (2009). The par-4-grp78 trail, more twists and turns. Cancer Biol. Ther..

[24] Zhao Y., Burikhanov R., Brandon J., Qiu S., Shelton B.J., Spear B., Bondada S., Bryson S., Rangnekar V.M. (2011). Systemic par-4 inhibits non-autochthonous tumor growth. Cancer Biol. Ther..

[25] Xiang W., Ke Z., Zhang Y., Cheng G.H., Irwan I.D., Sulochana K.N., Potturi P., Wang Z., Yang H., Wang J. (2011). Isthmin is a novel secreted angiogenesis inhibitor that inhibits tumour growth in mice. J. Cell Mol. Med..

[26] Zhang Y., Chen M., Venugopal S., Zhou Y., Xiang W., Li Y.H., Lin Q., Kini R.M., Chong Y.S., Ge R. (2011). Isthmin exerts pro-survival and death-promoting effect on endothelial cells through alphavbeta5 integrin depending on its physical state. Cell Death Dis..

[27] Chen M., Zhang Y., Yu V.C., Chong Y.S., Yoshioka T., Ge R. (2014). Isthmin targets cell-surface grp78 and triggers apoptosis via induction of mitochondrial dysfunction. Cell Death Differ..

[28] Chen M., Qiu T., Wu J., Yang Y., Wright G.D., Wu M., Ge R. (2018). Extracellular anti-angiogenic proteins augment an endosomal protein trafficking pathway to reach mitochondria and execute apoptosis in huvecs. Cell Death Differ..

[29] Cao Y., Chen A., An S.S., Ji R.W., Davidson D., Llinas M. (1997). Kringle 5 of plasminogen is a novel inhibitor of endothelial cell growth. J. Biol. Chem..

[30] Davidson D.J., Haskell C., Majest S., Kherzai A., Egan D.A., Walter K.A., Schneider A., Gubbins E.F., Solomon L., Chen Z. (2005). Kringle 5 of human plasminogen induces apoptosis of endothelial and tumor cells through surface-expressed glucose-regulated protein 78. Cancer Res..

[31] McFarland B.C., Stewart J., Hamza A., Nordal R., Davidson D.J., Henkin J., Gladson C.L. (2009). Plasminogen kringle 5 induces apoptosis of brain microvessel endothelial cells: Sensitization by radiation and requirement for grp78 and lrp1. Cancer Res..

[32] Nakatsuka A., Wada J., Iseda I., Teshigawara S., Higashio K., Murakami K., Kanzaki M., Inoue K., Terami T., Katayama A. (2013). Visceral adipose tissue-derived serine proteinase inhibitor inhibits apoptosis of endothelial cells as a ligand for the cell-surface grp78/voltage-dependent anion channel complex. Circ. Res..

[33] Fang S., Hong H., Li L., He D., Xu Z., Zuo S., Han J., Wu Q., Dai Z., Cai W. (2017). Plasminogen kringle 5 suppresses gastric cancer via regulating hif-1alpha and grp78. Cell Death Dis..

[34] Rondas D., Crevecoeur I., D’Hertog W., Ferreira G.B., Staes A., Garg A.D., Eizirik D.L., Agostinis P., Gevaert K., Overbergh L. (2015). Citrullinated glucose-regulated protein 78 is an autoantigen in type 1 diabetes. Diabetes.

[35] Misra U.K., Mowery Y., Kaczowka S., Pizzo S.V. (2009). Ligation of cancer cell surface grp78 with antibodies directed against its cooh-terminal domain up-regulates p53 activity and promotes apoptosis. Mol. Cancer Ther..

[36] Misra U.K., Pizzo S.V. (2010). Modulation of the unfolded protein response in prostate cancer cells by antibody-directed against the carboxyl-terminal domain of grp78. Apoptosis.

[37] de Ridder G.G., Ray R., Pizzo S.V. (2012). A murine monoclonal antibody directed against the carboxyl-terminal domain of grp78 suppresses melanoma growth in mice. Melanoma Res..

[38] Mo L., Bachelder R.E., Kennedy M., Chen P.H., Chi J.T., Berchuck A., Cianciolo G., Pizzo S.V. (2015). Syngeneic murine ovarian cancer model reveals that ascites enriches for ovarian cancer stem-like cells expressing membrane grp78. Mol. Cancer Ther..

[39] Lee A.S. (2005). The er chaperone and signaling regulator grp78/bip as a monitor of endoplasmic reticulum stress. Methods.

[40] Jakobsen C.G., Rasmussen N., Laenkholm A.V., Ditzel H.J. (2007). Phage display derived human monoclonal antibodies isolated by binding to the surface of live primary breast cancer cells recognize grp78. Cancer Res..

[41] Liu R., Li X., Gao W., Zhou Y., Wey S., Mitra S.K., Krasnoperov V., Dong D., Liu S., Li D. (2013). Monoclonal antibody against cell surface grp78 as a novel agent in suppressing pi3k/akt signaling, tumor growth, and metastasis. Clin. Cancer Res..

[42] Lee A.S. (2014). Glucose-regulated proteins in cancer: Molecular mechanisms and therapeutic potential. Nat. Rev. Cancer.

[43] Hensel F., Eckstein M., Rosenwald A., Brandlein S. (2013). Early development of pat-sm6 for the treatment of melanoma. Melanoma Res..

[44] Rasche L., Duell J., Morgner C., Chatterjee M., Hensel F., Rosenwald A., Einsele H., Topp M.S., Brandlein S. (2013). The natural human igm antibody pat-sm6 induces apoptosis in primary human multiple myeloma cells by targeting heat shock protein grp78. PLoS ONE.

[45] Rauschert N., Brandlein S., Holzinger E., Hensel F., Muller-Hermelink H.K., Vollmers H.P. (2008). A new tumor-specific variant of grp78 as target for antibody-based therapy. Lab. Invest..

[46] Rasche L., Duell J., Castro I.C., Dubljevic V., Chatterjee M., Knop S., Hensel F., Rosenwald A., Einsele H., Topp M.S. (2015). Grp78-directed immunotherapy in relapsed or refractory multiple myeloma - results from a phase 1 trial with the monoclonal immunoglobulin m antibody pat-sm6. Haematologica.

[47] Rasche L., Menoret E., Dubljevic V., Menu E., Vanderkerken K., Lapa C., Steinbrunn T., Chatterjee M., Knop S., Dull J. (2016). A grp78-directed monoclonal antibody recaptures response in refractory multiple myeloma with extramedullary involvement. Clin. Cancer Res..

[48] Arap M.A., Lahdenranta J., Mintz P.J., Hajitou A., Sarkis A.S., Arap W., Pasqualini R. (2004). Cell surface expression of the stress response chaperone grp78 enables tumor targeting by circulating ligands. Cancer Cell.

[49] Miao Y.R., Eckhardt B.L., Cao Y., Pasqualini R., Argani P., Arap W., Ramsay R.G., Anderson R.L. (2013). Inhibition of established micrometastases by targeted drug delivery via cell surface-associated grp78. Clin. Cancer Res..

[50] Staquicini D.I., D’Angelo S., Ferrara F., Karjalainen K., Sharma G., Smith T.L., Tarleton C.A., Jaalouk D.E., Kuniyasu A., Baze W.B. (2018). Therapeutic targeting of membrane-associated grp78 in leukemia and lymphoma: Preclinical efficacy in vitro and formal toxicity study of bmtp-78 in rodents and primates. Pharmacogenomics J..

[51] Kao C., Chandna R., Ghode A., Dsouza C., Chen M., Larsson A., Lim S.H., Wang M., Cao Z., Zhu Y. (2018). Proapoptotic cyclic peptide bc71 targets cell-surface grp78 and functions as an anticancer therapeutic in mice. EBioMedicine.

